# High-salt intake alters autonomic maturation trajectories assessed by an HRV-based ANS Age Index in rats

**DOI:** 10.3389/fphys.2026.1889830

**Published:** 2026-07-07

**Authors:** Sung Jun Hong, Dokyoon Kim, Taekyung Kim, Jeonghun Kim, ChangHwa Oh, Hye-won Lee, Jinyoung Youn, Young-Min Shon

**Affiliations:** 1 Biomedical Engineering Research Center, Samsung Medical Center, Seoul, Republic of Korea; 2 Smart Healthcare Institute, Samsung Medical Center, Seoul, Republic of Korea; 3 Department of Digital Health, Samsung Advanced Institute for Health Sciences and Technology (SAIHST), Sungkyunkwan University, Seoul, Republic of Korea; 4 AI Research Center, Samsung Medical Center, Seoul, Republic of Korea; 5 Department of Medical Device Management and Research, SAIHST, Sungkyunkwan University, Seoul, Republic of Korea; 6 Department of Neurology, Samsung Medical Center, Sungkyunkwan University School of Medicine, Seoul, Republic of Korea

**Keywords:** ANS Age Index, autonomic maturation, autonomic nervous system, heart rate variability, high-salt diet, model-derived trajectory, rat model, VGG16

## Abstract

Excessive dietary salt intake is known to affect autonomic and cardiovascular regulation, but its longitudinal impact on autonomic maturation remains poorly understood. This study developed a heart rate variability (HRV)-based autonomic nervous system (ANS) Age Index to quantify deviations from the normative trajectory of autonomic maturation in rats exposed to different levels of salt loading. Eighteen rats were assigned to control, 4% NaCl, or 8% NaCl diet groups and followed over an 11-week observation period. Three-lead electrocardiograms were recorded twice weekly for 5 min, and HRV-derived time-frequency representations were generated using short-time Fourier transform. Baseline-referenced HRV matrices were segmented using a 30-s sliding window with a 10-s shift, and each HRV segment was combined with zero-padded baseline-referenced body weight-change information to construct a 407 × 300 input for a VGG16-based regression model. The model was trained exclusively on control-group data to learn the normative autonomic maturation trajectory and was then validated using leave-one-animal-out cross-validation to reduce the risk of information leakage from non-independent augmented segments. In this animal-level validation, all augmented segments from one control animal were held out during model training in each fold. After averaging the 28 segment-level predictions within each ECG recording, the model achieved a recording-level root mean square error (RMSE) of 0.758 weeks, mean absolute error (MAE) of 0.608 weeks, and coefficient of determination (R²) of 0.943. After validation, a final control-trained model was applied to the high-salt groups to estimate model-derived deviations from the control-derived autonomic trajectory. The 4% NaCl group showed a sustained positive shift in ANS Age Index, indicating an older-than-expected model-predicted autonomic profile relative to the control-derived trajectory. In contrast, the 8% NaCl group showed persistently lower and flattened predicted autonomic age trajectories, indicating a younger-than-expected or altered model-derived autonomic trajectory. These findings suggest that high-salt intake may be associated with non-linear deviations in model-predicted autonomic trajectories. The proposed HRV-based ANS Age Index should be interpreted as an exploratory computational proxy for detecting diet-associated shifts in HRV-derived autonomic patterns, rather than as a validated physiological biomarker of autonomic aging.

## Introduction

The autonomic nervous system (ANS) is a major regulator of cardiovascular homeostasis, metabolic adaptation, and physiological resilience across growth and aging (Patural et al., 2019; Piantoni et al., 2021). Because autonomic regulation changes dynamically over time, the concept of autonomic maturation has emerged as a potentially informative index of biological development beyond simple chronological age. Heart rate variability (HRV), which reflects beat-to-beat variation in cardiac rhythm driven by sympathetic and parasympathetic modulation, has been widely used as a noninvasive biomarker of autonomic function (Task Force of the European Society of Cardiology and the North American Society of Pacing and Electrophysiology, 1996; Shaffer and Ginsberg, 2017). Conventional HRV analyses based on predefined time-domain and frequency-domain indices have provided important physiological insights; however, such summary measures may not fully capture the complex, nonlinear, and time-varying patterns embedded in longitudinal autonomic signals, particularly during development and in response to dietary or environmental perturbations (Hayano and Yuda, 2019; Agorastos et al., 2023; Olivieri et al., 2024).

Among the environmental factors known to influence autonomic and cardiovascular regulation, excessive salt intake is of particular interest. High-salt exposure has been associated with altered sympathetic activity, blood pressure variability, and neurogenic mechanisms contributing to cardiovascular dysregulation (McNeely et al., 2008; Stocker et al., 2013; Simmonds et al., 2014; Gomes et al., 2017; Larson et al., 2017; Dampney et al., 2018). Experimental studies have shown that excess dietary sodium can exaggerate sympathetic reflex responses even in normotensive animals, whereas chronic sodium loading beginning early in life may promote maladaptive cardiovascular regulation later in adulthood (Stocker et al., 2013; Simmonds et al., 2014; Gomes et al., 2017). These observations suggest that salt loading influences not only hemodynamic status but also the neural control systems responsible for cardiovascular adaptation (Larson et al., 2017; Dampney et al., 2018). Nevertheless, the longitudinal effect of different levels of salt exposure on the maturation trajectory of autonomic function remains poorly understood. In particular, it is unclear whether moderate and severe salt loading induce a similar biological response or whether they differentially shift model-predicted autonomic trajectories relative to a control-derived reference pattern.

Recently, biological age modeling based on physiological signals has emerged as a useful strategy for quantifying deviation from normal developmental or aging trajectories. In neuroimaging and electrophysiology, the difference between model-predicted biological age and expected chronological age has been proposed as a biologically meaningful marker of advanced or delayed physiological status. This concept has been successfully applied to sleep EEG, where deep learning-based brain age estimation has been shown to capture deviations from normative brain aging (Sun et al., 2019; Yook et al., 2022). Inspired by this framework, we reasoned that a similar approach could be extended from the brain to the autonomic nervous system. In parallel, our previous work demonstrated that HRV time-frequency representations can be effectively learned using a VGG16-based deep learning framework, supporting the feasibility of image-based modeling of autonomic dynamics.

In the present study, we aimed to estimate autonomic age in rats using a deep learning model trained on HRV-derived time-frequency representations augmented with longitudinal body weight change. Specifically, the HRV time-frequency image was combined with a baseline-referenced body weight-change row to form an integrated input representation, allowing the model to incorporate both autonomic dynamics and somatic growth-related information. When trained only on data from normally fed rats, the model was expected to learn the normative trajectory of autonomic maturation across the 11-week observation period. Applying this control-trained model to rats exposed to 4% and 8% NaCl diets allowed us to quantify the extent to which high-salt intake shifted autonomic physiology away from the normal developmental trajectory. In this context, the difference between model-estimated autonomic age and chronological week was defined as the ANS Age Index, which was used to assess whether salt loading was associated with directionally distinct model-derived deviations from the control-derived HRV trajectory.

Accordingly, the aim of the present study was to develop a control-trained VGG16-based age prediction model using HRV time-frequency representations augmented with baseline-referenced body weight change in rats maintained on a normal diet across an 11-week observation period, and to apply this model to rats exposed to 4% and 8% NaCl diets. Using the difference between model-predicted autonomic age and chronological week as an ANS Age Index, we sought to determine whether distinct levels of salt exposure differentially alter the longitudinal trajectory of autonomic maturation. We hypothesized that different levels of salt exposure would produce distinct deviations in model-predicted autonomic trajectories relative to the control-derived reference pattern. Specifically, we expected that the 4% NaCl group would show a positive ANS Age Index, corresponding to an older-than-expected model-predicted autonomic profile, whereas the 8% NaCl group would show a qualitatively different, lower or flattened model-derived trajectory. Under this framework, the ANS Age Index was used as an exploratory computational index of deviation from the control-derived HRV trajectory, rather than as a direct measure of biological autonomic aging.

## Methods

### Animals and experimental design

A total of 18 rats were included in this study and assigned to three dietary groups: a control group fed a standard diet (n = 6), a 4% NaCl high-salt diet group (n = 6), and an 8% NaCl high-salt diet group (n = 6). All animals were 6 weeks old at baseline. Baseline body weight was comparable among the three groups, with no statistically significant difference observed before dietary exposure. The baseline characteristics of the experimental animals are summarized in **Table 1**.

**Table 1:** Baseline characteristics of experimental animals.

Variable	Control	4% NaCl	8% NaCl	p-value
Number of animals, n	6	6	6	–
Age at baseline, weeks	6	6	6	–
Body weight, g	359.2 ± 12.6	354.3 ± 9.3	372.8 ± 18.2	0.087

Values are presented as mean ± standard deviation unless otherwise indicated. Statistical comparison of baseline body weight among the three groups was performed using one-way analysis of variance (ANOVA). A p-value < 0.05 was considered statistically significant.

The observation period lasted 11 weeks. Electrocardiogram (ECG) signals were recorded twice weekly in each animal, yielding 22 longitudinal measurement points, and each recording lasted 5 min with LabScribe software (iWorx Systems, Dover, NH, USA). Body weight was also measured longitudinally throughout the study period. Thus, repeated autonomic and growth-related data were acquired over the 11-week observation period to characterize the developmental trajectory of autonomic maturation under normal and high-salt dietary conditions.

The control group was used to establish the normative developmental trajectory of autonomic function across the experimental period. The 4% and 8% NaCl groups were subsequently used as post-training application groups to determine whether moderate and severe salt loading altered the predicted autonomic maturation pattern relative to the control-derived reference trajectory.

All procedures involving animal experimentation were approved by the Institutional Animal Care and Use Committee of the Samsung Medical Center, Sungkyunkwan University School of Medicine (approval no.: 20230817001).

### ECG acquisition and preprocessing

For each ECG recording, signal preprocessing was performed using the same analytical framework as in our previous HRV deep learning study. Briefly, a 3rd-order Butterworth high-pass filter with a cutoff frequency of 1 Hz was applied to reduce baseline drift and low-frequency noise related to respiration or movement. A 3rd-order Butterworth low-pass filter with a cutoff frequency of 15 Hz was used to suppress high-frequency noise, including muscle-related artifacts. In addition, a 6th-order Butterworth notch filter was applied to remove power-line interference. Consecutive R-peaks were detected using the Pan–Tompkins algorithm (Pan and Tompkins, 1985). Ectopic beats were corrected using a 20% filter, and polynomial detrending was applied to remove low-frequency trends from the HRV signal. To improve spectral estimation, the inter-beat interval series was cubically interpolated at 10 Hz.

### HRV time-frequency image generation using STFT

After ECG preprocessing, the inter-beat interval series was cubically interpolated at 10 Hz to generate an evenly sampled HRV signal. Because each ECG recording lasted 300 s, the interpolated HRV signal consisted of 3000 time points for each recording. To represent dynamic changes in autonomic regulation over time, short-time Fourier transform (STFT) was applied to the interpolated HRV signal (Elsenbruch et al., 2000).

The STFT converted the HRV signal into a two-dimensional time-frequency representation, in which the vertical axis represented frequency components and the horizontal axis represented temporal progression within the 5-min ECG recording. The resulting HRV time-frequency matrix had a dimension of 406 × 3000, corresponding to 406 frequency bins and 3000 temporal bins. This full-length HRV time-frequency representation was used as the basis for subsequent baseline-referenced normalization and sliding-window segmentation.

### Baseline-referenced bin-wise normalization

To reduce inter-individual variability in absolute HRV spectral power and to emphasize longitudinal changes from the initial autonomic state, baseline-referenced bin-wise normalization was performed. For each animal, the HRV time-frequency image at week 0 was used as the baseline reference, and each frequency-time bin at a given week was normalized to the corresponding baseline bin as follows:


$$Normalized\;HRV=\;\frac{HRV_{week\;x}-\;HRV_{week\;0}\;}{HRV_{week\;0}}$$

where HRV_week x_ represents the HRV power value at a given frequency-time bin at week x, and HRV_week0_ represents the corresponding baseline value at week 0. This procedure generated a relative HRV change map representing deviation from baseline across the experimental period. The normalization was performed before segmentation and therefore preserved the original full-length matrix structure of 406 × 3000.

### Sliding-window segmentation-based input augmentation

To increase the number of training samples while preserving the temporal structure of each ECG recording, sliding-window segmentation was applied to the baseline-normalized HRV time-frequency matrix. Each 5-min recording corresponded to 300 s, or 3000 temporal bins after 10-Hz interpolation. A 30-s sliding window was moved along the recording with a 10-s shift. Because each 30-s window contained 300 temporal bins, each segmented HRV time-frequency sample had a dimension of 406 × 300.

This segmentation strategy was used as an input augmentation procedure. Instead of treating each 5-min recording as a single sample, multiple temporally shifted HRV segments were generated from the same recording, allowing the model to learn local variations in autonomic dynamics across the recording period. A total of 28 partially overlapping segments were generated from each 5-min ECG recording. All augmented segments derived from the same recording retained the same chronological week label as the corresponding source recording.

### Construction of the combined HRV–body weight input

To integrate autonomic dynamics with longitudinal growth-related information, baseline-referenced body weight change was incorporated into the HRV-based input representation. For each animal, body weight change was calculated relative to the baseline measurement at week 0:

ΔBody weight = body weight at a given week − body weight at week 0

For each augmented 406 × 300 HRV time-frequency segment, the corresponding Δbody weight value was assigned according to the experimental week of the source ECG recording. Because body weight change is a single scalar value rather than a time-varying signal, it was encoded as a zero-padded auxiliary row. Specifically, a 1 × 300 body weight feature row was generated, in which the Δbody weight value was placed at a predefined position and the remaining elements were set to zero. This sparse encoding preserved the scalar nature of body weight change while allowing it to be integrated into the same image-like input format as the HRV representation.

**Figure 1:**
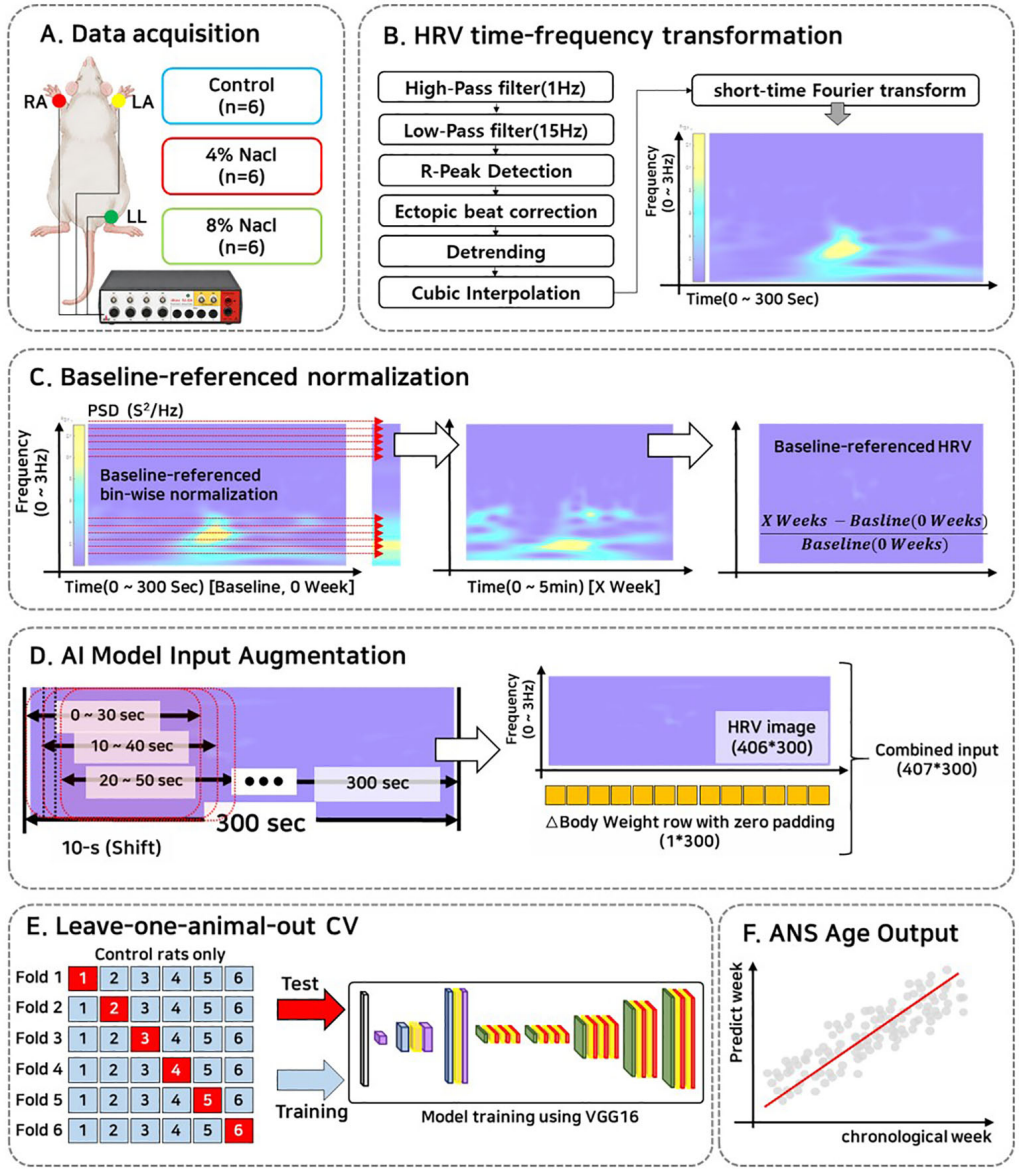
Overview of the HRV-based ANS Age Index modeling pipeline. Three dietary groups of rats were studied longitudinally: control, 4% NaCl, and 8% NaCl groups. Repeated three-lead ECG recordings and body-weight measurements were obtained during the 11-week observation period. ECG-derived inter-beat interval signals were converted into HRV time–frequency representations and normalized relative to week 0 to generate baseline-referenced HRV images. Each 300-s ECG recording was segmented using a 30-s sliding window with a 10-s shift, yielding 28 segments per recording. Each 406 × 300 HRV image was combined with a zero-padded 1 × 300 row encoding baseline-referenced body-weight change, resulting in a 407 × 300 model input. A VGG16-based regression model was trained using only control-group data and validated using leave-one-animal-out cross-validation. Segment-level predictions were averaged at the recording level, and a final control-trained model was then applied to the 4% and 8% NaCl groups. The ANS Age Index was defined as predicted week minus chronological week.

The zero-padded body weight row was appended to the bottom of the HRV time-frequency segment. Thus, each final model input consisted of a 406 × 300 HRV time-frequency image and a 1 × 300 zero-padded body weight-change row, yielding a combined input matrix of 407 × 300. This combined representation was designed to incorporate both HRV-derived autonomic dynamics and longitudinal body growth information for deep learning-based week prediction. The overall preprocessing, input construction, model validation, and ANS Age Index workflow are illustrated in **Figure 1**.

### Control-trained VGG16 age prediction model

A VGG16-based convolutional neural network was adapted for regression analysis to estimate the experimental week from the combined HRV–body weight input (Simonyan and Zisserman, 2014). The model input consisted of the final 407 × 300 matrix generated from the preceding preprocessing steps, and the target output was the corresponding chronological week of the recording. Unlike a classification model, the final output layer was configured to generate a continuous predicted week value.

The model was trained exclusively using data from the control group to learn the normative trajectory of autonomic maturation under standard dietary conditions. No data from the 4% NaCl or 8% NaCl groups were used during model training, model optimization, or cross-validation. The high-salt groups were used only for post-training application of the control-derived reference model.

ECG was recorded twice weekly over the 11-week observation period, yielding 22 longitudinal measurement points for model development after preprocessing and signal quality control. For model development, the control dataset was constructed from 6 rats across these 22 measurement time points, with 28 augmented segments generated from each 5-min ECG recording. Thus, the control dataset consisted of 3,696 input samples.

To reduce the risk of information leakage caused by overlapping augmented segments derived from the same animal or recording, model performance was evaluated using leave-one-animal-out cross-validation within the control group. In each fold, all augmented segments from one control animal were held out as the test set, and the model was trained using all segments from the remaining five control animals. This procedure was repeated six times so that each control animal served once as the held-out test animal. This validation strategy ensured that no augmented segments from the held-out animal were included in model training. Prediction performance was assessed using root mean square error, mean absolute error, and coefficient of determination.

Because each 5-min ECG recording generated 28 overlapping augmented segments, prediction performance was summarized at the recording level by averaging the predicted week values from all segments derived from the same ECG recording. After leave-one-animal-out cross-validation, a final VGG16 regression model was trained using all available control-group samples and was then applied to the 4% NaCl and 8% NaCl groups.

### Definition of ANS Age Index and application to high-salt groups

After development of the control-trained VGG16 regression model, the model was applied to the 4% NaCl and 8% NaCl groups using the same preprocessing and input construction procedures. For each augmented input sample, the model generated a continuous predicted week value. Because the model was trained only on control-group data, the predicted week was interpreted as the autonomic age of each sample relative to the normative autonomic maturation trajectory observed under standard dietary conditions.

To quantify deviation from the expected chronological trajectory, the ANS Age Index was defined as the difference between the model-predicted week and the actual chronological week of the corresponding ECG recording:

ANS Age Index = Predicted week−Chronological week

A positive ANS Age Index indicated that the input pattern was mapped by the control-trained model to a later point along the learned reference trajectory than expected for the actual experimental week. In contrast, a negative ANS Age Index indicated that the input pattern was mapped to an earlier point along the learned reference trajectory. Thus, the ANS Age Index was interpreted as a model-derived deviation metric rather than as a direct measure of biological autonomic aging or maturation.

All augmented segments generated from the same ECG recording retained the same chronological week label. The ANS Age Index was first calculated for each augmented input sample. For longitudinal visualization and statistical analysis, the 28 segment-level predictions derived from the same ECG recording were averaged to obtain one recording-level value per animal at each measurement point.

### Statistical analysis

Baseline body weight was compared among the control, 4% NaCl, and 8% NaCl groups using one-way analysis of variance. Values are presented as mean ± standard deviation for baseline animal characteristics.

For longitudinal analysis of the ANS Age Index, segment-level model predictions were first averaged within each 5-min ECG recording to obtain one recording-level predicted week value per animal at each measurement point. The ANS Age Index was then calculated as the difference between the recording-level predicted week and the corresponding chronological week. Because repeated measurements were obtained from the same animal across the experimental period, linear mixed-effects models were used to account for within-animal correlation. Fixed effects included group, week, and the group × week interaction, and animal identity was included as a random intercept.

To identify the specific time points at which each high-salt group diverged from the control-derived normative trajectory, time-point-specific control-referenced contrasts were performed within the mixed-effects framework. At each measurement point, the 4% NaCl and 8% NaCl groups were separately compared with the control group. The estimated group difference was calculated as the high-salt group value minus the control group value. Therefore, a positive estimate indicated a higher ANS Age Index than the control group, whereas a negative estimate indicated a lower ANS Age Index than the control group.

Because multiple week-specific comparisons were performed across the longitudinal experiment, p-values from all control-referenced contrasts were adjusted using the Benjamini–Hochberg false discovery rate procedure (Benjamini and Hochberg, 1995). Statistical significance was defined as a two-sided FDR-adjusted p-value < 0.05. Statistical analyses were performed using MATLAB with the Statistics and Machine Learning Toolbox. For visualization of longitudinal trajectories, data are presented as group mean ± standard deviation calculated from animal-level measurement-point means.

## Results

### Baseline characteristics of experimental animals

A total of 18 rats were included in the analysis, with 6 animals assigned to each dietary group: control, 4% NaCl, and 8% NaCl. All animals were 6 weeks old at baseline. Baseline body weight did not significantly differ among the three groups (control, 359.2 ± 12.6 g; 4% NaCl, 354.3 ± 9.3 g; 8% NaCl, 372.8 ± 18.2 g; one-way ANOVA, p = 0.087), indicating that the experimental groups were comparable before dietary exposure (**Table 1**).

### Performance of the control-trained VGG16 age prediction model

The VGG16-based regression model was trained using only control-group data to learn the normative trajectory of autonomic maturation. The control dataset consisted of 3,696 input samples generated from 6 rats across 22 longitudinal measurement time points, with 28 augmented segments derived from each 5-min ECG recording.

To address the non-independence of overlapping augmented segments, model performance was evaluated using leave-one-animal-out cross-validation within the control group. In each fold, all segments from one control animal were excluded from training and used only for testing, whereas segments from the remaining five control animals were used for model training. In this animal-level validation, after averaging the 28 segment-level predictions within each ECG recording, the model achieved a recording-level RMSE of 0.758 weeks, MAE of 0.608 weeks, and R² of 0.943. These results provide a more conservative estimate of model performance at the animal level because no augmented segments from the held-out animal were included in model training.

After completion of leave-one-animal-out validation, a final model was trained using all control-group samples and applied to the 4% NaCl and 8% NaCl groups to compare longitudinal model-predicted autonomic age trajectories across dietary conditions.

### Longitudinal trajectories of model-predicted autonomic age across dietary groups

**Figure 2:**
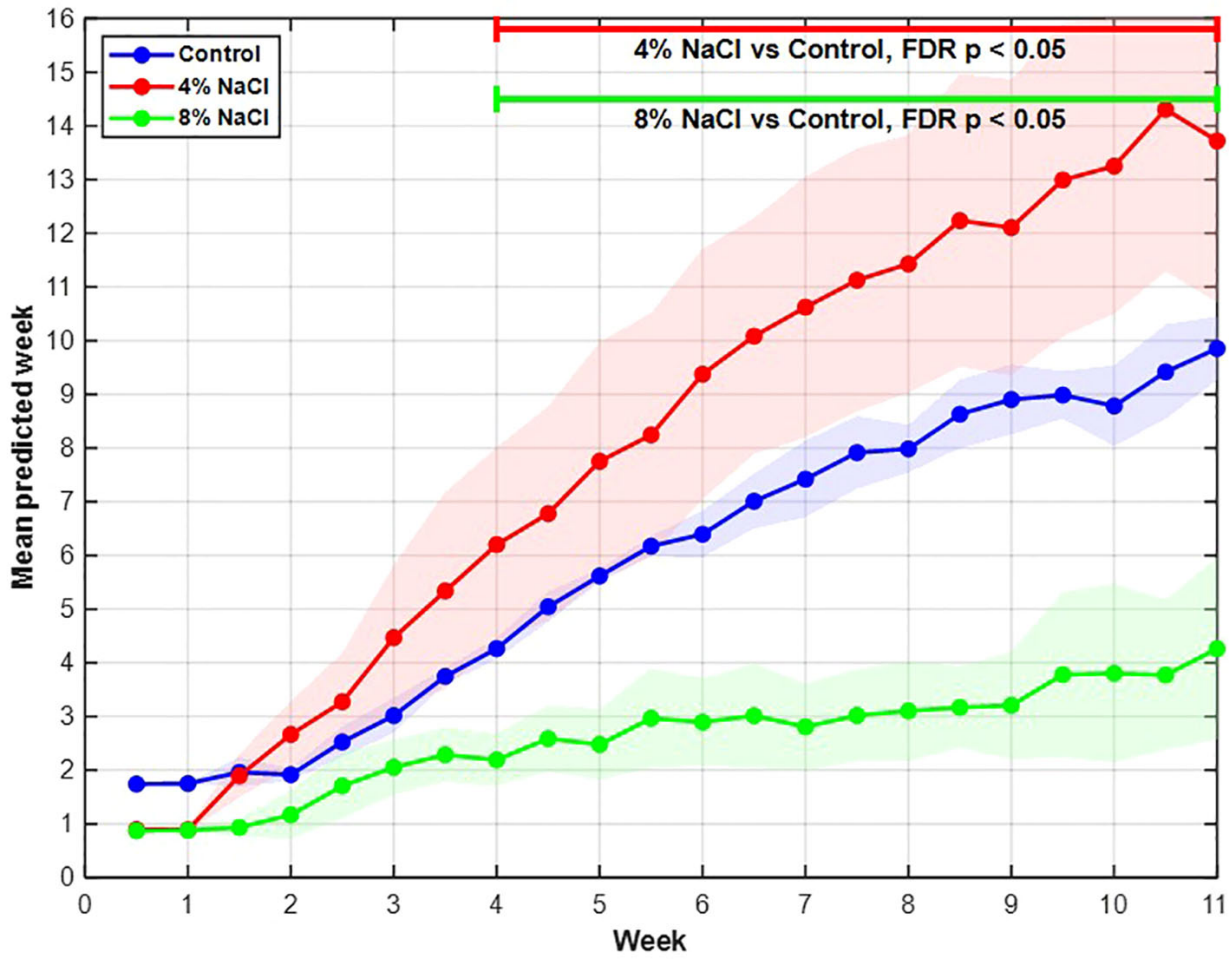
Longitudinal trajectories of model-predicted autonomic age across dietary groups. Control-group predictions were obtained using leave-one-animal-out cross-validation, in which all augmented segments from one control animal were held out during model training. For the high-salt groups, predictions were generated using the final model trained on all control animals. Segment-level predictions were averaged at the recording level, and values are presented as mean ± standard deviation across animals at each time point. The 4% NaCl group showed a higher model-predicted autonomic age trajectory than the control group, whereas the 8% NaCl group showed a lower and relatively flattened trajectory. Red and green horizontal bars indicate time intervals during which the 4% NaCl and 8% NaCl groups, respectively, significantly differed from the control group after FDR correction. Both high-salt groups showed significant divergence from the control-derived trajectory from week 4.0 onward.

The longitudinal trajectories of model-predicted autonomic age are shown in **Figure 2**. In the control group, the predicted week progressively increased over the 11-week observation period, consistent with the normative autonomic maturation trajectory learned from control animals. In contrast, the 4% NaCl group showed a steeper increase in predicted week and consistently higher predicted autonomic age values than the control group. Notably, the predicted values in the 4% NaCl group exceeded the corresponding chronological study week during the later phase of the experiment, suggesting an older-than-expected model-predicted autonomic profile.

Conversely, the 8% NaCl group showed markedly lower predicted week values than the control group throughout the observation period. The trajectory of the 8% NaCl group remained relatively flattened compared with both the control and 4% NaCl groups, suggesting a younger-than-expected or altered model-derived autonomic trajectory. In **Figure 2**, markers represent group-level mean predicted week values at each measurement point, and shaded regions indicate ± standard deviation. Horizontal bars indicate the time intervals during which each high-salt group significantly differed from the control group based on FDR-adjusted control-referenced contrasts. Together, these findings indicate that moderate and severe salt exposure were associated with distinct deviations from the control-derived model-predicted autonomic trajectory, with the 4% NaCl group showing an older-than-expected model-predicted autonomic profile and the 8% NaCl group showing a lower and relatively flattened model-derived autonomic trajectory.

### Time-point-specific divergence of ANS Age Index from the control trajectory

To determine when the high-salt groups significantly diverged from the control-derived normative trajectory, time-point-specific control-referenced contrasts were performed using linear mixed-effects models with FDR correction. The estimated contrast was defined as the high-salt group value minus the control group value; therefore, positive estimates indicate a higher ANS Age Index than the control group, whereas negative estimates indicate a lower ANS Age Index. The full statistical results, including estimates, standard errors, test statistics, raw p-values, FDR-adjusted p-values, and significance status, are provided in **Supplementary Table 1**.

After FDR correction, both the 4% NaCl and 8% NaCl groups significantly diverged from the control-derived model-predicted trajectory from week 4.0 onward. The 4% NaCl group showed positive contrasts relative to the control group, indicating a sustained shift toward an older-than-expected model-predicted autonomic profile. In contrast, the 8% NaCl group showed negative contrasts relative to the control group, indicating a lower and relatively flattened model-derived autonomic trajectory. These time-point-specific comparisons support the divergent longitudinal patterns shown in **Figure 2**, and the significant intervals are indicated by the horizontal bars.

## Discussion

In the present study, we developed an HRV-based ANS Age Index to quantify deviations from the normative trajectory of autonomic maturation in rats exposed to different levels of dietary salt loading. A VGG16-based regression model trained exclusively on control-group data captured HRV-derived temporal patterns within the control dataset. When this control-derived reference model was applied to the high-salt groups, 4% and 8% NaCl exposure produced directionally distinct model-predicted deviations. The 4% NaCl group showed a sustained positive shift in ANS Age Index after the early phase, corresponding to an older-than-expected model-predicted autonomic profile, whereas the 8% NaCl group showed persistent negative deviations and a relatively flattened model-derived trajectory. These findings should be interpreted as evidence of salt-associated shifts in HRV-derived autonomic patterns rather than as direct evidence of accelerated or delayed biological autonomic aging.

Previous studies have shown that high-salt intake can alter autonomic and cardiovascular regulation through sympathoexcitatory and neurogenic mechanisms. Stocker and colleagues summarized evidence that excess NaCl can influence central neural circuits involved in sympathetic outflow and cardiovascular control. Simmonds et al. further demonstrated that dietary salt intake exaggerated sympathetic reflex responses and increased blood pressure variability even in normotensive rats (Simmonds et al., 2014). In addition, Gomes et al. reported that chronic high-sodium intake after weaning could lead to neurogenic hypertension in adult Wistar rats, suggesting that early-life sodium exposure can have long-lasting effects on autonomic and cardiovascular regulation (Gomes et al., 2017). Experimental and review evidence further supports the involvement of hypothalamic and central autonomic circuits in salt-related sympathoexcitation and cardiovascular dysregulation (Larson et al., 2017; Dampney et al., 2018). Human data also indicate that dietary sodium can influence HRV-related autonomic regulation (McNeely et al., 2008). These previous studies collectively indicate that salt loading affects not only vascular or hemodynamic status but also the neural regulatory systems that control cardiovascular adaptation.

Our findings extend these observations by shifting the focus from isolated autonomic or hemodynamic outcomes to the longitudinal trajectory of autonomic maturation. Conventional high-salt studies often evaluate sympathetic tone, reflex sensitivity, blood pressure variability, or endpoint cardiovascular outcomes. In contrast, the present study used HRV time-frequency dynamics and deep learning-based age prediction to estimate whether the autonomic profile of each animal appeared older or younger than expected relative to a control-derived developmental trajectory. This approach allowed high-salt-induced autonomic dysregulation to be interpreted as a deviation in model-derived autonomic trajectory, rather than only as an increase or decrease in a single physiological parameter.

The positive ANS Age Index observed in the 4% NaCl group may be interpreted as an older-than-expected model-predicted autonomic profile under moderate salt loading. Because the present study did not directly measure sympathetic nerve activity, parasympathetic function, catecholamine levels, baroreflex sensitivity, or central autonomic circuit activity, the mechanistic meaning of the positive ANS Age Index remains inferential.

A sustained older-than-expected model-predicted autonomic profile may be consistent with altered HRV-derived autonomic regulatory patterns under moderate salt loading. However, because direct autonomic measurements were not performed, this interpretation remains hypothesis-generating. This interpretation is in line with prior evidence that dietary salt can exaggerate sympathetic reflexes and promote neurogenic cardiovascular dysregulation (McNeely et al., 2008; Stocker et al., 2013; Simmonds et al., 2014; Gomes et al., 2017; Larson et al., 2017; Dampney et al., 2018). From this perspective, moderate high-salt exposure may not simply alter HRV features transiently, but may be associated with a sustained older-than-expected model-predicted autonomic profile (Piantoni et al., 2021; Olivieri et al., 2024).

In contrast, the 8% NaCl group showed a persistently lower predicted autonomic age and a flattened trajectory compared with the control group. This pattern suggests that more severe salt exposure may not produce a simple dose-dependent positive shift in model-predicted autonomic age. Instead, the 8% NaCl group appeared to remain below the control-derived reference trajectory throughout the observation period. This finding may indicate a qualitatively different alteration in HRV-derived autonomic dynamics under severe salt loading. However, this should not be interpreted as direct evidence of delayed biological maturation. Thus, the 8% NaCl response may represent a qualitatively different form of autonomic dysregulation rather than a more severe version of the 4% NaCl pattern.

This non-linear pattern is biologically important. If high-salt exposure acted only as a monotonic stressor, one might expect the 8% NaCl group to show an even greater positive ANS Age Index than the 4% group. However, the opposite pattern was observed. This suggests that moderate and severe salt loading may be associated with different HRV-derived trajectory patterns. Moderate and severe salt exposure may therefore be associated with qualitatively different HRV-derived trajectory deviations, although the underlying physiological mechanisms remain to be determined.

In this framework, both positive and negative deviations from the control trajectory may be considered meaningful model-derived departures from the reference pattern, but their physiological significance requires further validation.

The present study also extends the broader concept of biological age modeling to the autonomic nervous system. In neuroimaging research, the difference between predicted biological age and chronological age has been used as a marker of advanced or delayed brain aging (Cole and Franke, 2017). Similar approaches have been applied to sleep EEG, where predicted brain age or neuroelectrophysiological age indices can capture deviations associated with brain aging, structural abnormalities, or sleep-related dysfunction (Sun et al., 2019; Yook et al., 2022). Our study applies this conceptual framework to HRV-derived autonomic dynamics. By defining the gap between model-predicted autonomic age and chronological week as the ANS Age Index, we provide a quantitative model-derived index of autonomic trajectory deviation. This approach may be particularly useful when physiological changes are complex, nonlinear, and difficult to summarize using conventional HRV indices alone (Hayano and Yuda, 2019; Agorastos et al., 2023; Olivieri et al., 2024).

Importantly, the ANS Age Index should be interpreted as a computational proxy derived from model-predicted deviation, not as a validated physiological biomarker of autonomic aging. Because the index is influenced by model architecture, HRV time-frequency feature construction, baseline-referenced normalization, sliding-window segmentation, and inclusion of body weight-related information, its biological meaning remains inferential. A positive or negative ANS Age Index indicates that the HRV-derived input pattern was mapped by the control-trained model to a later or earlier point along the learned reference trajectory. Therefore, the observed deviations may reflect salt-associated shifts in HRV dynamics rather than true acceleration or delay of biological aging.

A strength of this study is that the model was trained only on control-group data, allowing the control-derived trajectory to serve as a normative reference. The high-salt groups were then evaluated relative to this reference trajectory, rather than being used to train the model. This design enabled the ANS Age Index to function as a deviation metric, conceptually similar to brain age gap approaches. In addition, the use of HRV time-frequency representations allowed the model to capture dynamic autonomic information across time and frequency domains, while baseline-referenced normalization emphasized longitudinal changes from each animal’s initial physiological state.

Several limitations should be considered. First, the sample size was relatively small, with six animals per group, and the findings should be validated in larger independent cohorts. Second, although leave-one-animal-out cross-validation was used to reduce information leakage from overlapping augmented segments, the validation was still performed within the same experimental cohort and included only six control animals. Therefore, the reported model performance should be interpreted as an internal animal-level validation estimate rather than as evidence of external generalizability. Future studies should validate the model in larger independent datasets. Third, body weight change was incorporated into the model input to reflect longitudinal growth-related information; however, this variable may partially encode chronological progression and should be evaluated in future ablation analyses comparing HRV-only and HRV–body weight models. Fourth, the ANS Age Index is an indirect computational marker derived from HRV dynamics and body weight change, and it does not directly identify the underlying sympathetic or parasympathetic mechanisms. Additional mechanistic measures, such as direct sympathetic nerve activity, pharmacological autonomic blockade, catecholamine levels, baroreflex sensitivity, or central autonomic circuit analysis, would help clarify the biological basis of the observed age-index shifts. Finally, although the present study focused on autonomic maturation, future studies should examine how the ANS Age Index relates to cardiovascular, renal, metabolic, and behavioral outcomes under chronic salt loading.

In summary, the present findings suggest that high-salt intake is associated with distinct deviations from a control-derived model-predicted autonomic trajectory. The 4% NaCl group showed an older-than-expected model-predicted autonomic profile, whereas the 8% NaCl group showed a lower and relatively flattened model-derived trajectory. These results suggest that high-salt exposure may not produce a simple linear dose-dependent pattern, but may instead be associated with distinct HRV-derived autonomic trajectory deviations depending on exposure severity. The proposed HRV-based ANS Age Index provides an exploratory quantitative framework for detecting deviations from normative autonomic trajectory patterns, but its biological meaning requires further validation with larger samples, independent external validation cohorts, ablation analyses, and direct physiological measurements.

## Conclusion

In this study, we developed an HRV-based ANS Age Index using a control-trained VGG16 regression model to quantify model-derived deviations from a normative autonomic trajectory in rats exposed to different levels of dietary salt loading. The model provided an exploratory reference trajectory based on HRV-derived autonomic patterns observed in normally fed rats, against which salt-associated deviations in the high-salt groups could be evaluated.

When applied to the high-salt groups, the ANS Age Index showed two directionally distinct model-derived patterns. The 4% NaCl group showed a sustained shift toward an older-than-expected model-predicted autonomic profile, whereas the 8% NaCl group showed a persistently lower and relatively flattened model-derived trajectory. These findings suggest that different levels of salt exposure may be associated with distinct, non-linear shifts in HRV-derived autonomic patterns relative to a control-derived reference trajectory.

Overall, the present results support the potential utility of the ANS Age Index as an exploratory computational framework for detecting diet-associated deviations from normative autonomic trajectory patterns. However, the ANS Age Index should not be interpreted as a validated physiological biomarker of autonomic aging. Further studies with larger sample sizes, independent external validation cohorts, ablation analyses, and direct physiological measurements are needed to clarify the biological meaning and translational relevance of this model-derived index.

## Data Availability

The raw data supporting the conclusions of this article will be made available by the authors, without undue reservation.

## Supplementary material

Supplementary Table 1 Time-point-specific control-referenced contrasts of ANS Age Index. Estimates represent the difference between each high-salt group and the control group at each measurement point. Positive estimates indicate higher ANS Age Index values than the control group, whereas negative estimates indicate lower ANS Age Index values. P-values were adjusted using the Benjamini–Hochberg false discovery rate procedure.
